# Religious zeal as an affective phenomenon

**DOI:** 10.1007/s11097-020-09664-4

**Published:** 2020-03-13

**Authors:** Ruth Rebecca Tietjen

**Affiliations:** Department of Philosophy, Heinrich-Heine-Universität Düsseldorf, Universitätsstraße 1, 40225 Düsseldorf, Germany

**Keywords:** Religious zeal, Love, Anger, Passion, Emotion, Affective phenomenon, Religious violence, Fanaticism

## Abstract

What kind of affective phenomenon is religious zeal and how does it relate to other affective phenomena, such as moral anger, hatred, and love? In this paper, I argue that religious zeal can *be* both, and be *presented* and *interpreted* as both, a love-like passion and an anger-like emotion. As a passion, religious zeal consists of the loving devotion to a transcendent religious object or idea such as God. It is a relatively enduring attachment that is constitutive of who the zealot is, and it expresses itself in a distinctive set of mental and behavioral dispositions. Most importantly, it motivates uncompromising actions and involves intense, hot, and deep emotions. As an anger-like emotion, religious zeal is an occurrent affective state of mind that is intentionally directed towards a specific (immanent) object, characteristically a person or group of persons. It condemns the violation of a religious norm that is taken to be of absolute validity and general applicability. It motivates an action aiming at vengeance and retaliation, and it involves intense and hot feelings of hostility towards its object. I argue that rather than reducing the complex phenomenon of religious zeal to one of these two manifestations, we should reflect upon the question of how the two distinct conceptualizations relate to each other (and are interwoven with political interests).

## Introduction

Religious zeal is often mentioned in the context of discussions on religiously motivated or legitimized violence. It is supposed to motivate, be partly constitutive of, or even be synonymous with, religious fanaticism. Since religious fanaticism constitutes a pressing problem today, understanding the nature of religious zeal is not only of theoretical value but also of practical importance. Yet, despite its alleged significance and topicality, there is a striking lack of general philosophical and psychological theories of (religious) zeal (for an exception, see Olson 2007). Particularly, although religious zeal can intuitively be classified as an affective phenomenon, little attention has been paid to it in contemporary philosophy of emotion. This is even more surprising, as the tremendous amount of attention affective phenomena have received in the past few decades also has started to cover the domain of religion (Coakley 2012; Corrigan 2008; Lemmens and van Herck 2008; Roberts 2007; Wynn 2005). In providing an analysis of the phenomenon of religious zeal, my paper aims at contributing to fill this gap. Using the concept of ‘affective phenomena’ as an umbrella term covering emotions, moods, feelings, passions, and the like, I develop an account of what kind of affective phenomenon religious zeal is and how it relates to cognate affective phenomena, such as moral anger, hatred, and love.^1^

In developing a theory of religious zeal, two pitfalls must be avoided. First, we should not mistake the negative connotation the concept of religious zeal bears today in western media discourse as something inherent to the phenomenon itself (Olson 2007; Toscano 2017). Today, in a context where rationality is accepted as guiding value, and religion and the passions are regarded as ‘the other’ of reason, classifying others as religious zealots serves the function of criticizing them, discrediting them, and excluding them from discourse (Cavanaugh 2012). On the contrary, in the first century AD, the ancient Greek concept ζηλωτής at least occasionally was used as an honorific title (Elliott 2008). As Martin Hengel argues in his seminal *The Zealots*, one plausible explanation for the fact that the ancient historian Josephus refrained from calling the ‘fourth philosophy’ by their self-given name consists in the fact that he refused the positive connotation of their self-description. Rather than describing them as a religiously motivated liberation movement, he presented them as “lawless bandits and criminals” (Hengel 1989, p. 45). This shows how dependent the connotation of the concept of religious zeal is on the context within which it is used. Moreover, even within Christianity, it is common to distinguish ‘wrong,’ ‘false,’ ‘misguided,’ or ‘blind’ from ‘true,’ ‘godly,’ or ‘holy’ zeal (Edwards 2009, pp. 352–353).

That said, we also should not take violent forms of religious zeal to be merely derivative from peaceful ones, as nothing but abusive exceptions. It is necessary to be sensitive to the fact that religious zeal always already bears a violent potential, as is indicated by the paradigmatic biblical narrative about human zeal: the narrative about Phinehas in the Book of Numbers. Contrary to religious fanaticism – which is characterized by its inherent intolerance and proneness to violence (Katsafanas 2019) –, I take religious zeal to be an inherently ambiguous phenomenon (for a synonymous use of ‘fanaticism’ and ‘zealotry’, see Olson 2007 and Toscano 2017). It can “animate(…) dedication to truth, love, and beauty” but also “fuel militant religious and political conflicts with devastating social consequences” (McGregor 2006, p. 343). It is this ambiguity that partly explains the discomforting nature of religious zeal.

The second pitfall to be avoided is that we must be careful not to de-theologize religious zeal by focusing on its socio-political motivation only (Hengel 1989; Deines 2011), even if religious motives are often entangled with socio-political ones (Assmann 2016). Moreover, although the individual and socio-psychological conditions under which religious zeal is developed are crucial for understanding the phenomenon itself, we must be careful not to pathologize religious zeal by treating it as an ‘abnormal’ reaction (Sloterdijk 2008). This might makes us lose sight of the all too human nature of religious zeal.^2^ The same applies to the culturalization of religious zeal that essentializes it by treating it as a manifestation of a specific culture (Toscano 2017, p. xix) rather than as one possible expression of the human quest for the infinite, absolute, and unconditional (Ricœur 1986).

In the following, I defend the claim that religious zeal can *take* both the form of a love-like passion and the form of an anger-liker emotion. Moreover, it can be *presented* and *interpreted* as both a love-like passion and an anger-like emotion. Thereby, I exclusively address religious zeal that occurs in the context of the three Abrahamic monotheist religions without thereby wanting to deny the possibility and existence of religious zeal outside of this narrow context. Accordingly, I use the concept ‘religious’ to qualify experiences, beliefs, and practices that relate to the divine, holy, and unconditional, and more precisely to the belief in one (personal) God.^3^ There are two alternative ways of spelling out the relation between religious zeal as a love-like passion and religious zeal as an anger-like emotion: starting with the notion of religious zeal as a love-like passion and only then turning to violent and political forms of religious zeal as *one* possible expression of the love-like devotion to God; or the other way around, starting with our narrower everyday understanding of religious zeal as a necessarily violent and political phenomenon, and then widening our perspective by turning to the possible passionate foundation of the phenomenon. Both approaches are accompanied by characteristic dangers. Whereas the second approach is in danger of overestimating our incomplete and one-sided everyday understanding, the first one is at risk of starting with a philosophical theory that is all too remote from this understanding. In awareness of these risks, here, I follow the first approach, thereby highlighting the necessity to go beyond our everyday understanding – without denying its importance for our philosophical theorizing and its (restricted) legitimacy.

Accordingly, the structure of the paper is as follows. In section two, I introduce the basic conceptual distinction between emotions and passions. In section three, I outline a theory of religious zeal as a love-like passion. In section four, I outline a theory of religious zeal as an anger-like emotion. In section five, I reflect on the question of how both manifestations of religious zeal relate to each other, defending the claim that, despite their differences, we should still discuss both kinds of religious zeal together rather than apart. I conclude by arguing that we should be careful not to reduce the complex phenomenon of religious zeal to one of its possible expressions or forms.

## Emotions and passions

Following a major line of contemporary analytic philosophy of emotion, I use the concept of emotion to denote occurrent affective states of mind that are intentionally directed at specific objects in the world (Deonna and Teroni 2012; Goldie 2009; Helm 2001; Nussbaum 2001; Roberts 2003). (1) As occurrent affective states of mind, emotions have a characteristic phenomenality; it *feels* a specific way to have an emotion. Anger, for example, is claimed to involve “some level of felt hostility or antipathy towards its object” (Pettigrove 2012, p. 358). Herein, emotions differ from non-affective states of mind but also from emotional dispositions. (2) Emotions differ from mere bodily feelings, such as feeling one’s heartbeat raising or one’s body trembling, because emotions primarily are “feeling towards”: they are intentionally directed *towards* specific objects in the world (Goldie 2009). Whereas bodily feelings are a form of *consciousness of* one’s own body, bodily states, or the shift of one’s bodily states, emotions can be *about* objects, states of affairs, or properties other than the body (although they can also be *about* the body as well).^4^ We are angry *at* something or *with* someone, just as we love something or can be annoyed with ourselves. The potential *about-ness* of emotions, therefore, must be understood broadly, covering not only material objects but also fictional entities such as Anna Karenina or transcendent objects such as God (Crane 2001). In being intentionally directed towards specific objects in the world, emotions differ from moods that are commonly construed as affective mental states that are intentionally directed towards unspecific objects (or the world *as a whole)*, not intentionally directed at all, or pre-intentional (Goldie 2009; Heidegger 2006; Nussbaum 2001; Ratcliffe 2008). More precisely, emotions are evaluative states of mind. Emotions, such as anger and love, can be individuated (among other things) in virtue of their ‘formal object,’ i.e. the value property they ascribe to their intentional object. Anger, for example, is said to involve the evaluation “that its object (a) has wrongfully harmed someone or something of value or (b) has failed to care about someone or something in the appropriate way” (Pettigrove 2012, p. 357). (3) The evaluation in question is based on the person’s concerns (Roberts 2003), i.e. upon that which she attributes import, worth, and/or value to (Helm 2001). Being concerned with something means caring about and being affectively attached to an object. It involves a distinctive set of mental and behavioral dispositions. Typically (although not necessarily), in concerns, cognitive and conative dimensions are interfused with each other. They are neither pure value judgments held without any affective involvement nor pure desires devoid of all cognitive claims (Helm 2001). (4) Emotions involve characteristic action tendencies that may be constitutive of the emotion type in question but also purely contingent. Anger, for example, is claimed to be partly constituted by “the desire to lash out at its object or to see that object hurt” (Pettigrove 2012, p. 358). (5) Finally, although I presented the four characteristics consecutively, I take it that they are inextricably entangled with each other and can only be understood under mutual reference to each other (Goldie 2009).

Contrary to emotions, passions are not concern-*based* states of mind. They are themselves concerns. More precisely, they are specific kinds of concerns; namely, concerns that are characteristic of the person (Roberts 2007). They are part of her practical identity (Korsgaard 1996; Frankfurt 1988). Herein, they differ from emotions. Although emotions may relate to our practical identity because they are based on what we care about, they do not do so necessarily because not all our concerns are part of our practical identity. Moreover, the temporal structure of emotions differs from that of passions. As occurrent affective states of mind, emotions are bound to the specific situation in which we find ourselves. Emotional dispositions may form a part of our character (Deonna and Teroni 2012) or personality (Goldie 2004), but not every occurrent emotion is an expression of a character or personality trait. On the contrary, passions are relatively enduring states of mind that bestow one’s life with continuity, coherence, and/or meaning. We can have a passion for any number of different things: art or philosophy, collecting stamps or playing computer games, aesthetical experiences or the idea of democracy, our child, friend, spouse, or God. Emotions and passions mutually relate to each other. As concern-based states of mind, emotions can be based on passions, and passions in turn involve dispositions to emotions (Roberts 2007).

## Religious zeal as a love-like passion

It has often been observed that the phenomenal character of affective states of mind is difficult to capture in the theoretical language of philosophy; more adequately, it is expressed in a metaphorical language (Goldie 2009). The metaphors that are most prominently used to describe the phenomenon of religious zeal are the metaphors of heat and flame. Religious zeal is “a flame,” “the heat and fervor” of a flame (Edwards 2009, p. 352); it is an “ardent devotion” to an ideal that “puts a fire in the zealot’s belly that she is willing to sacrifice for” (Olson 2007, pp. 688–689). These metaphors indicate that religious zeal is a *consuming* feeling, a feeling that engages the zealot *as a whole*. It is a feeling that involves moments of hot, intense, and deep emotional involvement, and it is a feeling that involves some willingness to sacrifice. All these elements, in turn, relate to the intentional structure of religious zeal: religious zeal is an ardent devotion to a transcendent religious object that is taken to be of ultimate significance. On my view, these claims can best be captured if we conceive of religious zeal as a love-like passion.

As a passion, religious zeal consists of an affective attachment to a transcendent religious object such as God. This object is treated as an object of *ultimate* concern. An ultimate concern is a concern that is not based on any other concerns; it is an underivative concern (Roberts 2003, p. 142). Spoken in terms of values, religious zeal involves an ascription of a final rather than of an instrumental value (Korsgaard 1983). Herein, it resembles love that commonly is conceptualized as an (affective) attitude in which we value someone (or something) for his or her own sake (Taylor 1975; Velleman 1999). The value in question cannot be relativized with reference to any prior or superior value. Moreover, the zealot is *wholeheartedly* committed to the object of his or her zealous devotion. His or her attachment to God is a *consuming* feeling that engages the zealot *as a whole*. A ‘shallow zealot’ is a contradiction in terms. The attachment in question is relatively enduring. As in the case of love, it is an essentially historic relationship between oneself (or a specific community of faith) and God (or another transcendent religious object) (Rorty 1987; Assmann 2018). Finally, like love, it is an identity-defining relationship (Frankfurt 1999, 2004; Helm 2012): a relationship that is partly constitutive of the zealot’s (individual or social) practical identity. It bestows his or her life with continuity, coherence, and/or meaning, and involves a distinctive set of mental and behavioral dispositions.

On the affective level, religious zeal as a love-like passion involves a disposition to intense, hot, and deep emotions. Whereas intensity is a measure for how strongly a feeling is felt, depth is a measure for how embedded an emotion is within a person’s web of mental states (Cataldi 1993; Pugmire 2007). Deep emotions are based on concerns that are relatively central to this web. States in the center are those that amount to a significant change in the web when they are changed or removed. Since the zealot is ultimately and wholeheartedly concerned with the object of his or her passionate devotion, he or she will respond with intense and hot emotions when the object in question is affected either positively or negatively. Herein, zealous evaluations differ from cold-hearted evaluations; i.e., from evaluations that lack any affective involvement but also from mediating, cautious, or tentative affective evaluations. Moreover, as a relatively enduring, identity-defining concern, religious zeal gives rise to *deep* emotions. As the zealot is wholeheartedly committed to his or her object of zealous devotion, so too are the emotions that are based on this devotion consuming and all-encompassing.

On the behavioral level, religious zeal as a love-like passion necessarily expresses itself in series of actions, in repeated actions, or in habitualized forms of behavior. One who fails to (regularly) express his or her passionate devotion in his or her actions or behavior will not count as a religious zealot. Thus, repetition and habituation account for the historicity and specific temporal structure of religious zeal as a love-like phenomenon that develops over time and bestows one’s life with continuity, coherence, and/or meaning. Actions motivated by religious zeal as a love-like passion are characterized by a threefold ‘uncompromisingness.’ First, in cases where one’s object of zealous devotion is affected, religious zeal does not allow for compromises regarding the question of whether to act at all*.* Evaluations based on one’s ultimate concern for God translate themselves into action immediately. However, despite this immediacy, zealous actions are not necessarily unreflective. While some of them are impulsive and spontaneous actions committed in the heat of passions, others are well-conceived and long prepared (so that rather than being accompanied by intense and hot feelings, they may even be performed cold-heartedly). Second, in cases where one’s object of zealous devotion is affected, religious zeal as a love-like passion does not allow for compromises regarding the question of *what* to do. The zealot is willing to employ any means. He or she is willing to make sacrifices in order to secure the well-being of what he or she infinitely cares about in passion. Third, religious zeal as a love-like passion does not allow for compromises regarding the way how the action is performed. The zealot acts wholeheartedly and invests all his or her willpower and energy. In its motivational force, religious zeal differs from half-hearted behavior, wherein we are not completely absorbed by our activity or are involved with several things at once. Such half-hearted behavior includes actions accompanied by a doubt about the value and meaning of what we do, actions making compromises for private or economic reasons, or akratic actions in which our behavior diverges from our practical judgment about what is best to do.

To summarize, we can say that rather than giving rise to specific emotions or specific actions, religious zeal as a love-like passion is best characterized by the fact that it disposes one to hold affective evaluations and conduct actions in a specific way; namely, wholeheartedly and uncompromisingly. The phenomenal heat, intensity, and depth, and the motivational force thereby mirror both the distinct way in which the zealot is concerned with his or her object of zealous devotion – ultimately and wholeheartedly – and the inherent relationship between passions and practical identity.

Depending on how structured and integrated the mind of the zealot is, religious zeal as a passionate phenomenon can occur in three different forms. First, the concern for God may be one concern among others. One zealously devotes oneself to God *within the context of specific recurring circumstances*. Think of a Christian going to church every Sunday morning, being fully immersed in the service, and not accepting any adverse circumstances as excuse for refraining from his holy duty. The repetition and habituation of his religious actions bestow his life with continuity. Yet, the relevance he attributes to religion remains restricted to a specific domain of his life. Second, one may zealously *devote one’s life to religion*. Think of the Desert Fathers who lived an ascetic life in the solitude of the desert. For them, religion was not (merely) a form of ritualized behavior as it is for the zealous congregant but was, rather, a form of life. In this sense, the relevance of religion was not restricted to one domain of one’s life among others. Instead, religion was taken to be an overarching principle relevant for all domains of one’s life. In cases like this, the concern for God is a master-concern. As such, it bestows one’s entire life not only with continuity but also with coherence. Yet, the relevance attributed to religion remains restricted to one’s own life. Third, one may zealously devote one’s life to religion and thereby conceive of religion as something total: as an overarching principle relevant not only for all domains of one’s own life but also for the lives of others. Zealots of this kind strive not only for religious self-transformation but also for social and political change. Accordingly, the devotion to God not only bestows their life with continuity and coherence but also with social and/or political meaning.

These three ‘aggregate states’ of religion can be interpreted as three forms of ‘totalization,’ ranging from a context-sensitive ‘totalization,’ to an all-encompassing ‘total religion’ (Assmann 2018). In the conceptual domain of fanaticism, a similar distinction can be found; namely, the distinction between ‘being a fan’ and ‘being a fanatic’ (Passmore 2003). What does this mean for the question of violence? As the aforementioned examples of the zealous congregant and the Desert Fathers illustrate, passionate religious zeal is not necessarily violent. Even when religious zeal occurs as ‘total religion,’ it need not necessarily express itself in acts of violent aggression. For example, one might refer to a Muslim who zealously devotes his life to God and aims at social and political transformation by putting mercy and compassion at center stage. Accordingly, whether passionate religious zeal gives rise to violence does not only depend on its degree of totalization but also on the content of the religious principles it is committed to. Nonetheless, all three forms of passionate religious zeal involve a violent potential and this potential increases with the degree of totalization – as does the promise of salvation. The zealous congregant goes to church no matter what other, non-religious duties he has to deny in doing so. The ascetics are willing to sacrifice their health or even their lives for their religious principles. Finally, the religiously motivated freedom fighter – at least under exceptional conditions – is willing to use ‘unconventional’ means in order to change the existing social and political order that he or she judges to be evil and/or unjust. Indeed, as I argued, religious zeal as a passionate phenomenon is partly defined by the fact that it involves a willingness to sacrifice. This willingness to sacrifice corresponds to the unconditional, wholehearted commitment that is characteristic of religious zeal as an evaluative phenomenon. Whereas less than total forms of religious zeal allow for non-religious principles to be on a par with one’s religious commitments, total conceptions of religion conceive of religious principles as all-encompassing and absolutely overriding. This explains why their violent potential is higher but also why their ‘positive’ transformative power is stronger, as it is demonstrated by religiously motivated peace-building processes (Appleby 2000). Moreover, the aforementioned examples demonstrate that the meaning of ‘violence’ and ‘sacrifice’ vary significantly. Violence may be directed against symbols, objects, or oneself rather than against others. Moreover, it may be seen as an exception rather than as a rule – and this makes an important difference.

## Religious zeal as an anger-like emotion

In the previous section, I have argued that religious zeal as a passionate phenomenon – which occurs in different degrees of totalization – is a love-like attachment to a transcendent religious object involving a distinctive set of mental and behavioral dispositions. Moreover, I have pointed out that even though religious zeal as a love-like passion involves a violent potential that increases with the degree of totalization, it is neither necessarily violent nor necessarily political. However, contemporary political and media discourse draws a different picture of religious zeal that cannot simply be ignored. What first comes to mind when considering the notion of religious zeal probably are not acts of benevolence but rather other-directed acts of violent aggression against people who are blamed for having committed a sacrilege. Contemporary examples include the attack on the French newspaper Charlie Hebdo in 2015 by the Al-Qaeda members Saïd and Chérif Kouachi, and the murder of the US-American physician John Bayard Britton by the Presbyterian anti-abortionist Paul Jennings Hill in 1994 (Olson 2011). But the association of religious zeal with religious violence is not an invention of the present age. For example, in the Book of Numbers chapter 25, we are told that God’s fierce anger against the Israelites was evoked by the fact that they began to have sexual intercourse with Moabite women and to worship the Baal of Peor, a Moabite God. Therefore, God commands to kill the culprits. Moses conveys to the Israelite judges the divine commandment to kill all followers of the Baal of Peor among the people of Israel. The Israelite Phinehas follows Moses’ call by driving a spear through his fellow believer Zimri and the Midianite Cozbi while they are having sexual intercourse. Consequently, the plague God had brought upon the people of Israel stops. As a reward, God offers Phinehas a covenant of peace and of lasting priesthood because he had been zealous for God. Examples like this fuel the still ongoing debate on whether (the Abrahamic) monotheist religions are inherently violent.

All the three aforementioned examples primarily present religious zeal as an anger-like emotion rather than a love-like passion. As an anger-like emotion, religious zeal is directed at a specific immanent object, characteristically a person or group of persons. It condemns the violation of a religious norm or law that is taken to be of absolute validity and general applicability. Furthermore, it motivates an action aiming at vengeance and retaliation. Finally, it involves a hot and intense feeling of hostility towards its object. I will now consider each of these elements in turn to gain a clearer understanding of what it means to conceive of religious zeal as an anger-like emotion rather than as a love-like passion.

First, religious zeal is presented as a phenomenon that is intentionally directed at a particular immanent object (a singular person or several persons) and consists of an affective evaluation that condemns the violation of a religious norm or law. As moral anger, religious zeal as an emotional phenomenon “involves a double reference – to a person or people and to an act” (Nussbaum 2016, p. 17). Whereas the target of religious zeal is a particular person or group of persons, its focus is a specific transgression of a religious norm that is attributed to the person and for which the person is held responsible (Sousa 1987). Yet, although religious zeal is evoked by a specific deed – the transgression of a religious norm by a responsible agent – it is still the person *as a whole* that is condemned. Something similar has claimed to be true of moral anger (Roberts 2003, p. 203; Nussbaum 2016, p. 49). This becomes particularly obvious in the fact that religious zeal paradigmatically involves the motivation to annihilate the other altogether rather than to ‘rectify’ the blameworthy action attributed to the other.

Second, as an emotional phenomenon, religious zeal is based on the commitment to a religious norm; for instance, the divine commandment not to worship any other Gods or the prescription not to depict and insult the prophet Muhammad. This norm is taken to be of absolute validity; i.e., is taken as a norm that cannot be suspended by reference to any other kinds of evaluative measures. This absolutely overriding power of the affective evaluation is characteristic of religious zeal as an emotional phenomenon and is partly constituted by its motivational force and its phenomenal heat, intensity, and depth. In the case of monotheistic religions, religious norms and laws paradigmatically are conceived to be of divine origin, so that the commitment to a particular religious norm can be interpreted as part and parcel of a more general attachment to God as an object of ultimate concern. This points us back to the discussion of religious zeal as a love-like passion in the previous section because the anger-like condemnation of the violation of a religious norm or law can be based on – and even partly constitutive of – the zealot’s passionate devotion to God. As such, I can be interpreted as ‘zeal *for* God.’^5^

The fact that the violent aggression is directed at a third party demonstrates that the religious norm in question is not only taken to be of absolute validity but also of general applicability. It applies not only to oneself but also, or even primarily, to others – whether a specific group of people or everyone. Moreover, the violent zealot not only holds religious norms to apply to other people, he or she also takes him- or herself to be in a position to judge others. Something similar is true of moral anger (Roberts 2003, p. 204). As we have seen, this is not necessarily the case for all forms of zealous devotion to God. As the examples of the fervent congregant and the Desert Fathers prove, religious zealots may conceive of religion as ritual or aim at religious self-transformation rather than at social or political change. So, it is the third, total form of zealous devotion to God that potentially expresses itself in and/or is constituted by other-directed acts of violent aggression. Accordingly, the examples in the beginning of this section describe zealots who take religious norms and laws to be of absolute validity and general applicability. They conceive of these norms and laws as norms and laws that cannot be outweighed by any other evaluative measures and that apply to everyone – or at least their own people –, ultimately placing themselves in a position to judge and punish others. Accordingly, their reaction expresses an understanding of religion as a way of life rather than as ritualized behavior. More precisely, it expresses an understanding of religion as a way of life demanded from everyone rather than just from chosen people. Furthermore, it expresses an understanding of religious principles as superordinate principles relevant for all other domains of life (as opposed to being coordinate or subordinate to other principles).

Anger-like religious zealotry can be straightforwardly explained as a reaction to the violation of religious norms. Focusing on cases of interreligious violence such as the violent attack on Charlie Hebdo, one can argue that the zealots in question take religious norms and laws to be universalizable (as it is commonly claimed about moral norms and laws) and, therefore, applicable even to those who did not profess Islam and live in a predominantly secular state. Moreover, one might point to the fact that, as in the case of moral norms, it is constitutive of the commitment to religious norms that one shows not only self-reflexive emotions that reflect to what extent oneself meets the norms in question but also other-directed emotions. Not only guilt (a moral emotion directed at a *first* person) and resentment (a moral emotion directed at a *second* person) but also indignation (a moral emotion directed at a *third* person) are constitutive of accepting a moral norm (Strawson 1962). Even more, one might argue that it is *particularly* the impersonal emotion of indignation that dignifies emotions as moral, whereas the personal emotion of resentment is more likely to be infused by non-moral, personal concerns (Strawson 1962). Applied to the case of religious zeal, this would mean that being disposed to feel anger-like religious emotions in the case of transgressions of religious norms is partly constitutive of the commitment to a religious norm that is taken to be of general applicability.

Focusing on cases of inner-religious violence such as the case of Phinehas, one might also argue that having and/or showing the emotions in question may be partly constitutive of the zealot’s (individual and/or social) practical identity. Phinehas might take the validity of religious norms and laws to be part and parcel of the covenant between God and the people of Israel, and, therefore, take their validity to be restricted to his own people. *If* Phinehas’ attachment to God is indeed an affective attitude of the kind described in the previous section – i.e., an affective attachment to an object that is partly constitutive of his practical identity (Korsgaard 1996; Velleman 2002; Ricœur 1986) –, then his reaction to Zimri’s act of idolatry not only tells us something about his value commitments but also about his practical identity itself. Moreover, *if* the collective identity of the people of Israel is indeed partly constituted by the affective and essentially historic relationship between them and God (Assmann 2018), then Zimri’s act of idolatry not only endangers his own identity but also the identity of the people of Israel as a whole. It is not only a violation of a legal covenant but also an assault on the identity-defining, affective, love-like relationship between God and the Israelites. Something similar applies to other religious communities, although the specific way the idea of collective identity is construed will depend on which religion and denomination we are dealing with. Although it *need not* be the case that there are individual or collective identity-defining passions involved in phenomena of religious violence, conceiving of them as such provides a further explanation for the eruptive force of religious violence. This already holds if the attitude in question is defining the zealot’s individual identity. Even more so, it does if the attitude is defining the zealot’s social identity or the collective identity of his or her community of faith. In this case, additionally to the idea of an identity-defining attitude, a social layer of explanation is introduced into our theory.^6^ To summarize, we can say that being disposed to feel anger-like religious emotions in the face of transgressions of religious norms might reflect the intimate relationship between the obedience to religious norms and (one’s conception of) individual and/or collective identity.

Whereas the first part of the explanation referring to the nature of religious commitments as such explains zealous reactions to idolatry no matter who committed them, the second part of the explanation referring to the love-like relationship between God and oneself or one’s community of faith particularly accounts for acts of aggressive violence against one’s fellow believers. As such, the two parts of the explanation point us to the more general distinction between inner- and interreligious violence (Assmann 2016, pp. 45–57; Collins 2003).

Third, the examples listed in the beginning of this section present the anger-like emotion of religious zeal not only as an evaluative and concern-based state of mind but also as a motivational phenomenon involving a desire for vengeance and retaliation. More precisely, they present it as a phenomenon that necessarily motivates actions. Again, we would not classify anyone as a religious zealot whose religious beliefs, desires, and emotions do not express themselves in his or her actions and behavior at all. In this way, religious zeal as an anger-like emotion differs from ordinary emotions. It is controversial whether emotions are *necessarily* motivational. Examples that at least question the claim that emotions are necessarily motivational include the fear of a baseball fan who is afraid that his favored team might lose the game but feels no inclination to intervene; the fear of an aviophobe in the airplane that represents the situation as one in which one is better off doing nothing at all; the fear of a soldier in the battle-field whose sense of professional duty not only overrides his motivation to flee but erases it altogether; or the art-horror of a moviegoer who does not feel any inclination to flee but rather enjoys feeling threatened (Davis 1987; Döring 2003; Goldie 2009; Tappolet 2010). Even if one concedes that (particular kinds of) emotions are necessarily motivational states of mind, usually the motivational force is not taken to be compelling. Anger, for example, is commonly claimed to be partly constituted by the desire for revenge or redemption (Aristotle 2018, 1378a-1378b; Kauppinen 2018; Nussbaum 2016, pp. 14–56). Yet, *taking* revenge is not constitutive of anger. Other motives may be stronger and other practical considerations more persuasive than the craving for revenge. The moral value of anger may even be dependent on the capability to overcome the desire for revenge and transform it into a more constructive form of engagement (Nussbaum 2016, pp. 35–40). In contrast, religious zeal as an anger-like emotion *necessarily* expresses itself in actions and/or behavior.

More precisely, as an anger-like emotional phenomenon, religious zeal is characterized by the same threefold ‘uncompromisingness’ that I claimed to be characteristic of religious zeal as a love-like passion. It necessarily translates itself into action. It asks for uncompromising action, and the actions in question are committed wholeheartedly, under investment of all one’s power of will and energy. However, religious zeal as an anger-like emotion motivates specific kinds of actions; namely, actions aiming at vengeance and retaliation. In this way, it resembles moral anger that involves a desire to punish one’s offender or to see him hurt (Pettigrove 2012, p. 358; Roberts 2003, p. 204; Nussbaum 2016, pp. 14–56). However, it differs from ‘mature’ forms of anger (or ‘quasi-anger’) that involve a desire for restoration, aim at insight and behavioral change on the side of the offender or generally aspire to social welfare (Kauppinen 2018; Nussbaum 2016; Roberts 2003, p. 221). Rather, religious zeal as an anger-like emotion aims at the destruction and annihilation of its target object and, therein, resembles hatred (Szanto forthcoming).

Fourth and finally, the aforementioned examples present religious zeal as an emotional phenomenon with a characteristic intensity and heat. The zealots in question *fervently* condemn and kill or murder those they blame for having committed a sacrilege. Their emotion involves an intense and hot feeling of *hostility* towards its object.

To summarize, we can say that as an anger-like emotion, religious zeal is intentionally directed at specific objects in the world and consists of an affective evaluation that condemns the violation of a religious norm that is taken to be of absolute validity and general applicability. It necessarily translates itself into action, motivates an action aiming at vengeance and retaliation, and involves an intense and hot feeling of hostility towards its object. All three dimensions thereby are mutually entangled with each other. The fact that the evaluation is taken to be of absolute validity is reflected by the unmediated way in which the evaluation translates itself into action. The unmediated way in which the evaluation translates itself into action expresses itself in the characteristic fervent feeling, and the fervent feeling in turn refers back to the absoluteness of one’s evaluation. As an anger-like emotion, religious zeal need not be based on an identity-defining concern. However, conceiving of it as an emotion that is based on an identity-defining attachment to God as an object of ultimate concern provides a further explanation of the heat and intensity, the violent ‘uncompromisingness,’ and the eruptive force of religious violence.

## Conclusions

Our everyday understanding suggests that religious zeal is an anger-like emotion that is intentionally directed at specific (immanent) objects in the world, characteristically a person or group of persons. It consists of an affective evaluation that condemns the violation of a religious norm that is taken to be of absolute validity and general applicability. It motivates an action aiming at vengeance and retaliation, and it involves hot, intense, and – in some cases – deep feelings of hostility towards its object. In this paper, I argued that we need to contrast this conceptualization of religious zeal as an anger-like emotion with a more encompassing, neutral conceptualization of religious zeal as a love-like passion. Doing so invites us to broaden, partially revise, and question our everyday understanding. First, religious zeal cannot only be understood as an emotion but also as a passion. As such, it consists of the ardent devotion to a transcendent religious object with which one is ultimately concerned. Second, religious zeal as a passionate phenomenon is not necessarily political. As the examples of the fervent congregant and the devoted ascetics striving for self-transformation prove, religious zealots may conceive of religion as ritual or aim at religious self-transformation rather than at social or political change. Third, religious zeal need not necessarily be a ‘negative’ and backward-looking phenomenon that condemns violations of religious norms and motivates actions aiming at vengeance and retaliation. On the contrary, it also might be a ‘positive’ and forward-looking phenomenon that involves feelings of empathy for the suppressed and love for the creation of God, and that motivates actions of benevolence and worship. Fourth, none of the three forms of passionate religious zeal is necessarily violent. Nonetheless, they all bear a violent potential and this potential increases with the degree of totalization – as does the promise of salvation. Finally, one may wonder whether the concept of religious zeal should not be used neutrally to denote a specific way of holding affective evaluations and conducting actions – namely wholeheartedly and uncompromisingly – rather than a specific kind of evaluation and a specific kind of action, or even positively to denote specific kinds of positive evaluations attributing sacred values and motivating actions aiming at salvation.

As a state of mind that can take both the form of an emotion and a passion, religious zeal resembles love (and hatred). Love can be a relatively enduring, affectionate attachment to a person that is characteristic of the loving person and involves a distinctive set of mental and behavioral dispositions; and it can be a specific kind of affective evaluation that is bound to a specific situation (Roberts 2003, pp. 284–297; Fischer et al. 2018). However, the conceptualization of emotional religious zeal as an anger-like phenomenon and the conceptualization of passionate religious zeal as a love-like phenomenon suggest that the difference between emotional and passionate religious zeal is greater than the difference between emotional and passionate love (and hatred). Not only are we dealing with different kinds of affective phenomena (emotion versus passion) but also with different affective types (anger versus love). This gives rise to the question of how both phenomena relate to each other and what it is that they share that motivates discussing both of them under the label of ‘religious zeal’ rather than keeping them separate.

First, although I pointed out that religious zeal as a love-like passion does not necessarily express itself in other-directed acts of violent aggression, as a form of unconditional commitment it is still partly defined by the willingness to sacrifice. As a passionate phenomenon, religious zeal disposes us to feel emotions and conduct actions in a specific way; namely, wholeheartedly and uncompromisingly. One possible expression and form that the willingness to sacrifice can take if religion is conceived as something ‘total’ is what I described as the anger-like emotion of religious zeal. Taking this extreme possibility into consideration is crucial for understanding the love-like passion of religious zeal itself. Second, although I pointed out that religious zeal as an anger-like emotion need not be a ‘deep’ emotion, conceiving of it as such – and, more precisely, conceiving of it as an emotion that is based on an identity-defining, ultimate concern – helps us to understand the eruptive force of the phenomenon. It helps us to understand the absoluteness of the evaluation, the intensity and heat of the feeling, and the ‘uncompromisingness’ of the action that are constitutive of religious zeal as an anger-like emotion. It does so even more if the identity in question is not just the believer’s individual identity but the identity of a religious community of faith. So, at the very least, reflecting on the possible passionate foundation of religious zeal as an anger-like emotion is crucial for gaining a full understanding of the phenomenon in question. This passionate foundation may consist of a love-like (total) devotion to God, but we may also wonder whether acts of violent aggression might not indicate a lack of wholehearted love-like commitment rather than its totalization or be an expression of passionate hatred rather than love.

These considerations indicate that we only can understand emotional and passionate religious zeal under mutual reference to each other. They are not necessarily two distinct phenomena; they can also be understood as two dimensions of one and the same phenomenon. Still, one might object that the concept of (passionate) religious zeal should be reserved for those forms of passionate devotion to God that conceive of religion as something total and express themselves in acts of violent aggression. Indeed, it is partly a question of convention and stipulative definition whether one uses the concept of (passionate) religious zeal in a narrow or wide sense. My analysis did not provide a conclusive argument for one or the other use; rather, it aimed at demonstrating that we can only gain a full understanding of religious zeal even in its most extreme, violent and aggressive forms if we conceive of it in continuity with – and contrast to – less extreme or even peaceful and amicable forms of religious engagement that are motivated by an ardent devotion to God.

Another important reason for discussing emotional and passionate forms of religious zeal together rather than separately consists in the fact that religious zeal cannot only take both the form of an emotion and the form of passion. It can also be presented and interpreted as such. Reducing religious zeal to either an anger-like emotion or a love-like passion implicitly serves different political purposes. If religious zeal is presented as an emotion, it appears as a reactive attitude; i.e., as an attitude that is a reaction to the specific situation the subject is in. If it is presented as a passion, it appears as a trait inherent to the person’s identity. By presenting religious zeal as an emotion, the idea of a religious conception of life is marginalized. By presenting religious zeal as a passion, the relevance of situational conditions can be downplayed; religious violence can be pathologized but also rationalized. These fragmentary considerations indicate that the presentation, interpretation, and conceptualization of religious zeal is always already entangled with the perspective of those offering the conceptual analysis and in danger of being misused for political purposes. Accordingly, a philosophical theory of religious zeal must not only be critical but also self-critical, and it should be particularly careful not to rashly reduce the complex phenomenon of religious zeal to one of its dimensions or forms. Particularly, it should neither regard religious zeal as *necessarily* violent nor should it conceive of violent forms of religious zeal as *nothing but* abusive exceptions.

## Footnotes

1 I first presented an account of religious zeal in Tietjen (forthcoming). There, I develop a theory of religious zeal by analyzing the biblical narrative about Phinehas. The article primarily is directed to a theological readership. It refers to philosophical and theological literature as well as to (German) everyday language. The present article provides a more elaborate philosophical analysis of religious zeal as an affective phenomenon. It makes use of a phenomenological methodology and is embedded in contemporary philosophy of emotion.

2 Contrary to Sloterdijk, I do not claim that there is a *necessary* relation between religion and violence. Rather, I agree with Kippenberg that the relation between (monotheistic) religions and violence is neither necessary nor impossible but contingent (Kippenberg 2011, p. 13).

3 The concept of religion is highly contested. See Pals 2015 for a discussion of nine theories of religion. For a more fine-grained analysis, it would be worthwhile to reflect on the question of how different understandings of religion influence our conception of religious zeal. However, due to limits of space, I will confine myself to providing a theory of religious zeal that is based on a presupposed substantial definition of religion. Similarly, the question of how religious zeal relates to political forms of zeal must be left open here for future research.

4 One’s own bodily conditions may themselves become the intentional object of one’s emotions (as it is the case when one is afraid of one’s raising heartbeat). However, there are two feelings involved in these cases: a bodily feeling (feeling one’s heartbeat raise) and a feeling towards (being afraid *of* one’s raising heartbeat). Moreover, one might argue that *as a consciousness of one’s own bodily conditions* bodily feelings are themselves intentional states of mind (Crane 1998). However, they still seem to lack the evaluative quality that commonly is taken to be characteristic of emotions.

5 In accordance with other prominent passages of the Pentateuch (most importantly the recitation of the Ten Commandments in Exodus 20:1–17 and Deuteronomy 5:4–21), the biblical narrative about Phinehas not only characterizes Phinehas as zealous but also (and primarily) God Godself. This points to another interpretation of what it means to be ‘zealous for God’; namely, feeling zealous with and/or in place of God (Tietjen forthcoming).

6 Going beyond my analysis, fanaticism is commonly claimed to be a ‘group phenomenon’; i.e., an *essentially* collective phenomenon (Crosson 2003; Katsafanas 2019; Marcel 2008). The question of whether religious zeal is a group phenomenon addresses only one of several possible dimensions of sociality. For example, one may also wonder how my claim that religious zeal is directed at *particular* persons who are blamed for *specific* norm transgressions relates to the claim that fanaticism has a group as its object and involves an essentialization and collectivization of its target object (Crosson 2003; Marcel 2008; Szanto forthcoming). This question is of great importance, but here it must be left open for future research.
